# Emergence and Evolutionary Response of *Vibrio cholerae* to Novel Bacteriophage, Democratic Republic of the Congo^1^

**DOI:** 10.3201/eid2812.220572

**Published:** 2022-12

**Authors:** Meer T. Alam, Carla Mavian, Taylor K. Paisie, Massimiliano S. Tagliamonte, Melanie N. Cash, Angus Angermeyer, Kimberley D. Seed, Andrew Camilli, Felicien Masanga Maisha, R. Kabangwa Kakongo Senga, Marco Salemi, J. Glenn Morris, Afsar Ali

**Affiliations:** University of Florida Emerging Pathogens Institute, Gainesville, Florida, USA (M.T. Alam, C. Mavian, T.K. Paisie, M.S. Tagliamonte, M.N. Cash, F.M. Maisha, M. Salemi, J.G. Morris, Jr., A. Ali);; University of Florida College of Medicine, Gainesville (C. Mavian, T.K. Paisie, M.S. Tagliamonte, M.N. Cash, M. Salemi, J.G. Morris, Jr.);; University of California, Berkeley, California, USA (A. Angermeyer, K.D. Seed);; Chan Zuckerberg Biohub, San Francisco, California, USA (K.D. Seed);; Tufts University School of Medicine, Boston, Massachusetts, USA (A. Camilli);; Appui Medical Integre aux Activites de Laboratoire (AMI-LABO), Goma, Democratic Republic of the Congo (R.K.K. Senga); University of Goma, Goma (R.K.K. Senga);; University of Florida College of Public Health and Health Professions, Gainesville (M.T. Alam, A. Ali)

**Keywords:** cholera, *Vibrio cholerae*, antimicrobial resistance, bacteria, viruses, bacteriophages, Democratic Republic of the Congo, United States

## Abstract

Cholera causes substantial illness and death in Africa. We analyzed 24 toxigenic *Vibrio cholerae* O1 strains isolated in 2015–2017 from patients in the Great Lakes region of the Democratic Republic of the Congo. Strains originating in southern Asia appeared to be part of the T10 introduction event in eastern Africa. We identified 2 main strain lineages, most recently a lineage corresponding to sequence type 515, a *V. cholerae* cluster previously reported in the Lake Kivu region. In 41% of fecal samples from cholera patients, we also identified a novel ICP1 (Bangladesh cholera phage 1) bacteriophage, genetically distinct from ICP1 isolates previously detected in Asia. Bacteriophage resistance occurred in distinct clades along both internal and external branches of the cholera phylogeny. This bacteriophage appears to have served as a major driver for cholera evolution and spread, and its appearance highlights the complex evolutionary dynamic that occurs between predatory phage and bacterial host.

Cholera remains an ongoing public health threat on the continent of Africa, especially in the Democratic Republic of the Congo (DRC), which in 2020 reported the largest number of cases (19,789) of any country in the world with the exception of Yemen (*1*). Biotype El Tor strains of the seventh cholera pandemic (P7ET) were first reported in Africa in the early 1970s (*2*–*6*). Major outbreaks occurred in DRC in 2008, 2009, 2011–2012, 2013, and 2015–2017; in 2017 alone, an estimated 53,000 cholera cases with 1,145 deaths were reported from 20 of 26 provinces in DRC (*2*). Outbreaks have been most persistent in the eastern part of DRC in the Great Lakes region, along the Albertine Rift (*2*–*4*).

As reported elsewhere (*3*), strains appear to have been initially introduced into this area as part of what has been characterized as the T5 introduction (1970–1972) under the first wave of P7ET. In 1992, as part of the third wave of P7ET, the disease was reintroduced by a strain from southern Asia, in what has been designated as the T10 introduction event (*3*). Subsequent studies have documented persistence of T10 strains in this region, and ongoing cholera outbreaks in the Great Lakes region and spread of strains from this area suggest establishment of a regional focus of endemic disease derived from the T10 introduction (*6*).

Bacteriophages (phages) and their host bacteria follow predator–prey dynamics that drive co-evolution, resulting in the long-term persistence of both within ecosystems (*7*). Phage predation has been linked with seasonal patterns of cholera emergence and with clinical response to infection in humans (*8*–*11*). Phages are generally highly specific to their host; thus, both phage and susceptible host must maintain a dynamic equilibrium to coexist. Recently, it has been shown that mobile genetic elements associated with sulfamethoxazole/trimethoprim (SXT) antimicrobial resistance, designated as SXT integrative conjugative elements (ICEs), can determine phage resistance in *V. cholerae* (*12*). Furthermore, another study has demonstrated that susceptibility to phage killing of marine *V.* *lentus* was mediated by as many as 6–12 mobile genetic elements (*13*). Taken together, these recent studies support the concept that phage/host in situ interplay has a major role in adaptation and evolution.

Using microbiologic, phylogenomic, and molecular clock analyses, we investigated endemic cholera in the DRC Great Lakes regional hotspot. We also explored the genetic resistance of these *V. cholerae* strains to a novel ICP1 (Bangladesh cholera phage 1) *V. cholerae* phage isolated in cholera patients in the region and genetically distinct from previous ICP1 phages detected in Asia (*14*,*15*). 

## Methodsphage/

### Isolation and Characterization of Toxigenic *V. cholerae* O1 and Virulent Phages

In an initial study involving the isolation and characterization of toxigenic *V. cholerae* O1 strains, we collected fecal samples from suspected cholera patients admitted to cholera treatment centers around Goma, DRC, during 2015–2017 (Table). After collection, we brought the samples to the Laboratoire Provincial de Sante Publique du Nord-Kivu in Goma for microbiological and serologic analysis. We isolated bacteria and confirmed species using methods described elsewhere (*16*), then stored strains in soft Luria-Bertani (LB) Miller agar (0.7% agar) and sent them to the Emerging Pathogens Institute at the University of Florida (Gainesville, FL, USA) for sequencing.

**Table:** Characteristics of toxigenic *Vibrio cholerae* O1 strains isolated from the Democratic Republic of the Congo, 2015–2017*

Strain	Isolation date	Province/location	Serotype		Susceptibility of *V. cholerae* to ICP1_2017_A_DRC†	Mutation in O1 antigen and other genes‡	SRA ID
			Ogawa	Inaba			
AGC-1	2015 Apr 30	North Kivu/Kirotshe	–	+	S	–	SRR15192533
AGC-2	2015 May 18	Goma/Buhimba	–	+	S	–	SRR15192532
AGC-3	2015 May 20	Mutwanga	–	+	R	*rfbD*	SRR15192521
AGC-4	2015 Mar 07	Goma/Buhimba	–	+	R	*rfbN*	SRR15192516
AGC-5	2015 Mar 20	Goma/Buhimba	–	+	S	–	SRR15192515
AGC-6	2015 Jul 26	Goma/Buhimba	–	+	R	*rfbV*, VC0559 (hypothetical), *rplE*, *phrA*, *fliD*, VC0672 (hypothetical)	SRR15192514
AGC-7	2015 Jun 06	Goma/Buhimba	–	+	S	–	SRR15192513
AGC-8	2015 Aug 06	Goma/Buhimba	–	+	S	–	SRR15192512
AGC-9	2016 Jun 20	Maniema/Kabambare	+	–	S	–	SRR15192511
AGC-10	2016 Aug 09	Karisimbi/Hop Millitaire	–	+	R	*rfbD*	SRR15192510
AGC-11	2016 May 28	Alimbongo	–	+	R	*rfbD*	SRR15192531
AGC-12	2016 Jul 27	South Kivu/Fizi	+	–	S	–	SRR15192530
AGC-13	2016 Aug 08	Maniema/Kimbilulenge	+	–	S	–	SRR15192529
AGC-14	2017 May 18	Kirotshe/Rubaya	–	+	S	–	SRR15192528
AGC-15	2017 May 31	Rutshuru/Hgr	–	+	S	–	SRR15192527
AGC-16	2017 Jun 10	Rutshuru/Hgr	–	+	S	–	SRR15192526
AGC-17	2017 Jul 01	Nyiragongo/Turunga	–	+	S	–	SRR15192525
AGC-18	2017 Jul 03	Goma/Hop.Provincial	–	+	S§	*manA*	SRR15192524
AGC-19	2017 Jul 03	Goma/Hop.Provincial	–	+	S	–	SRR15192523
AGC-20	2019 Jul 03	Goma/Hop.Provincial	–	+	S	–	SRR15192522
AGC-21	2017 Jul 06	Karisimbi/Prison centrale	–	+	S	–	SRR15192520
AGC-22	2017 Jul 14	Karisimbi/Majengo	–	+	S§	*manA*	SRR15192519
AGC-23	2017 Jul 19	Karisimbi/Majengo	–	+	R	*rfbB*	SRR15192518
AGC-24	2017 Jul 15	Karisimbi/Majengo	–	+	S	*rfbU*	SRR15192517

*R, resistant; S, susceptible; +, positive; –, negative †Susceptibility to a virulent ICP1 phage (ICP1_2017_A_DRC) determined by strains yielding either complete resistance or forming turbid plaques in response to phage infection in plaque assay. The penultimate column indicates which strains had mutations in the O1-antigen biosynthetic complex and in other genes in the chromosome, with the mutated gene designated. AGC-18, AGC-22, and AGC-24 sustained 1, 1, and 18 bp deletion mutations in the indicated gene(s), resulting in a frame shift mutation in that gene, but all other ICP1 phage-resistant isolates sustained >1 missense mutation in the O-antigen biosynthetic gene cluster. ‡As detected by analysis using single-nucleotide polymorphism, insertion/deletion, or both. §Plaques were turbid as described elsewhere (*29*).

In a second study, we tried to isolate phages preying on *V. cholerae* O1 strains from fecal samples obtained in 2016–2017 from 41 additional cholera patients. We centrifuged cholera rice-water fecal samples at 5,000 × *g* for 10 minutes and filtered resultant supernatant through a 0.22-μm syringe filter, stored them at 4°C in a sterile microfuge tube, and sent them to the Emerging Pathogens Institute for analysis. To identify virulent phages, we tested each filtered fecal sample using standard plaque assay against *V. cholerae* O1 AGC-15, a strain we randomly selected from the DRC isolates from the first part of the study (Table). AGC-15 has the wild-type *ompU* sequence, which encodes the receptor for ICP2; it also has the wild-type O1-antigen biosynthetic genetic region that serves as the receptor for ICP1 and ICP3 (*14*) and lacks any PLE elements mediating immunity to ICP1 (*17*). For phage purification, we picked a single clear plaque using a Pasteur pipette into 1 mL of LB broth and incubated it overnight at 4°C to enable the phage to diffuse out of the soft agar. We made high-titer stocks of purified phage by infecting AGC-15 with phage in LB broth culture.

### Whole-Genome Mapping and High-Quality Single-Nucleotide Polymorphism Calling

We performed whole-genome sequencing on the 24 *V. cholerae* O1 isolates from the first part of the study with the Illumina MiSeq for 500 cycles (Qui); we further conducted high-quality single-nucleotide polymorphism (hqSNP) calling (Appendix). The final genomewide hqSNP alignment included 120 T10 sublineage *V. cholerae* genome sequences: 24 strains collected as part of our study (Table); 71 from publicly available genomes from outbreaks in eastern DRC during 2014–2016 (*6*); 6 archival and publicly available DRC genomes collected during 2001–2013; 17 genomes collected across Africa during 1998–2014; and 2 publicly available genomes from India, ancestors of T10 sublineage (*3*) (Appendix Table 1). We performed multilocus sequence typing analysis using the online tool PubMLST (K. Jolley, unpub data, https://doi.org/10.12688/wellcomeopenres.14826.1) (Appendix Table 2).

### Phylogeography

All datasets used in this study passed phylogenetic quality checks (Appendix Figure 1). To explore the origins of strains in the eastern portion of DRC and neighboring countries we used the Bayesian phylogeographic coalescent-based method implemented in BEAST version 1.10.4 software (*18**–**20*). The reconstruction of *V. cholerae* O1 spatiotemporal spread from different locations through Bayesian phylogeography requires calibration of a molecular clock. We estimated evolutionary rates implementing a Hasegawa-Kishino-Yano nucleotide substitution model (*21*) with empirical base frequencies, gamma distribution of site-specific rate heterogeneity, and ascertainment bias correction (*22*), testing a constant demographic prior against nonparametric demographic models, Gaussian Markov random field Skyride (*23*) and Bayesian Skyline plot (*24*), to rule out spurious changes in effective population size inferred by a nonparametric model, which would, in turn, effect timing of divergence events (*25*). We obtained the weighted average of synonymous (*dS*) and nonsynonymous substitution rates (*dN*) in the protein-coding regions of the *V. cholerae* O1 genome for all internal and external branches from a subset of 200 Bayesian maximum credibility clade (MCC) trees randomly obtained from the posterior distribution of trees, as described elsewhere (*26*,*27*).

### Whole genome sequencing, genome assembly and annotation of DRC phages

We sequenced 8 plaque-purified phages isolated from 8 independent patient fecal samples with Illumina MiSeq for 50 cycles. We obtained >200-fold coverage that helped with de novo assembly of each phage genome into 1 complete contig using CLC Genomics Workbench (QIAGEN, https://www.qiagen.com). We manually confirmed and corrected low-coverage or problem areas as needed to ensure authentic genome assembly. We annotated phage genomes as described elsewhere (*15*) and deposited sequences into the National Center for Biotechnology Information Sequence Read Archive (BioProject identification no. PRJNA748018; Appendix Table 3). 

## Results

**Figure 1:**
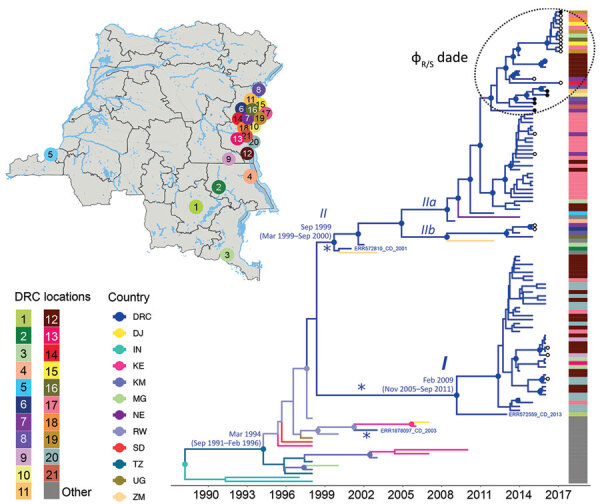
Spatiotemporal evolution and dissemination of *Vibrio cholerae* epidemic in the Democratic Republic of the Congo, 2015–2017. Sampling locations of *V. cholerae* strains sequenced in this study are indicated on the map. Each sampling location is coded by color and number, defined in the key; location colors are indicated for each tip in the maximum clade credibility tree as a heatmap (exact locations in Appendix Table 2). The tree was inferred from full genome *V. cholerae* isolates from DRC, neighboring countries, and Asia. Branches are scaled in time and colored by country of origin. Circles in internal nodes indicate posterior probability support >0.9, and the colors indicate ancestral countries inferred by Bayesian phylogeographic reconstruction. Circles at tips indicates the strains collected and sequenced in this study, with black circles designating phage-resistant strains. Notations *I*, *II*, *IIa*, and *IIb* indicate well-supported lineages and sublineages circulating in DRC during outbreaks. Asterisks (*) indicate potential spillover events within the Great Lakes region originated from neighboring countries. The tree with full tip labels is provided in Appendix Figure 2.

Of the 24 toxigenic *V. cholerae* O1 strains isolated from fecal samples from cholera patients attending cholera treatment centers during 2015–2017 in the Goma region, 21 (87.5%) were serotype Inaba and 3 (12.5%) serotype Ogawa (Table). All strains in wave 3 were *ctxB* genotype-I and within the T10 introductory clade (*3*,*6*). Consistent with findings published elsewhere (*3*), our MCC tree (Figure 1; Appendix Figure 2) indicated a mean time for the most recent common ancestor (tMRCA) of the T10 *V. cholerae* sublineage introduced to Africa of March 1994 (95% highest posterior density [HPD] September 1991–February 1996). In addition, our analysis showed that subsequent independent introductions (spillover events denoted by asterisks in Figure 1) in the DRC Great Lakes region likely occurred from Rwanda. The first spillover, in May 2001 (95% HPD September 1999–June 2001), is represented by a DRC isolate, ERR1878097_CD_2003, that branches out of a lineage circulating in Rwanda (Figure 1). The other event resulted in 2 major monophyletic clades that match multilocus sequence types reported elsewhere (*6*).

**Figure 2:**
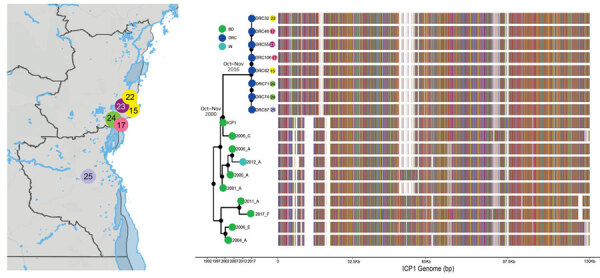
Bayesian inference of the phylogenetic relationship between phages and mutation patterns in the Democratic Republic of the Congo and ICP1 (Bangladesh cholera phage 1) patterns from Asia. Sampling locations of ICP1 strains from DRC are shown on the map. Each sampling location is coded by color and number, also indicated at the tip of the maximum clade credibility tree (exact locations in Appendix Table 2). The tree branches are scaled in time, and the circle tip points are colored by the location of origin, as indicated in the key. Circles in internal node indicate posterior probability support >0.9. To the right of the MCC tree, the genomic composition of each isolate is displayed: red, adenine; green, cytosine; yellow, guanine; and blue, thymine. White spaces indicate gaps at that location in the genome.

The tMRCA of the first major lineage (denoted as I in Figure 1), February 2009 (95% HPD November 2005–September 2011), corresponds to the sequence type 69 cluster (*6*) identified during the first reported outbreaks of cholera in DRC during 2008 and 2009. This cluster included the only Ogawa serotype isolates present in our collection. However, the long branch separating the isolate from the Tanganyika province (ERR572559_CD_2013) strain at the base of the monophyletic clade raises the possibility of unsampled *V. cholerae* strains (either from Rwanda or other neighboring countries) that could constitute missing linkage between this DRC lineage and its actual ancestor. The second major lineage (denoted as II in Figure 1) contains all of the Inaba serotype strains collected. According to the molecular clock calibration, lineage II tMRCA dates to September 1999 (95% HPD March 1999–September 2000). The monophyletic clade also includes 3 strains identified in Zambia and Niger, which were likely the result of spillover events from DRC. This clade further divides into 2 sublineages (IIa and IIb in Figure 1) that diverged in November 2004 (95% HPD September 2002–March 2007). Overall, molecular clock and phylogeographic reconstruction suggests circulation of cholera lineages in DRC years before the first reported cholera outbreaks in 2008–2009.

Phages were isolated from 17/41 (41.5%) fecal samples screened, on the basis of formation of plaques on strain AGC_15_CD_2017. Whole-genome sequencing of a subset (n = 8) of these phages showed that they shared high similarity in sequence (hqSNPs = 114) and diverged substantially (hqSNPs = 8,441) from the ICP1 phage isolated from Bangladesh and India (Figure 2). Previously, a total of 185 core open reading frames were identified as being conserved in ICP1 isolates collected over a 12-year period in Bangladesh and India (*15*). Uniquely, the DRC ICP1 lacks 15 of these core open reading frames and also has 10.6 kb of novel sequence in the first third of the genome. Of particular note, genomes do have the anti-*Vch*ind5 factor OrbA (*12*). However, like most ICP1-encoded gene products, most genes unique to DRC ICP1 are classified as hypothetical proteins because of a lack of an informative BLAST identification.

We screened our 24 DRC *V. cholerae* O1 strains for susceptibility to DRC ICP1 by plaque assays using ICP1_2017_A_DRC as a reference phage. Eighteen (75%) of the 24 *V. cholerae* strains were susceptible to ICP1_2017_A_DRC (Table); we observed that 2 strains had turbid plaques on plaque assay, as described elsewhere (*28*). At the genome level, all resistant strains, and 3 of 6 sensitive strains, including the 2 strains that produced turbid plaques, carried >1 mutation in genes that belong to the O1-antigen biosynthetic gene cluster. ICP1 uses the O1 antigen as its receptor, and *V. cholerae* is known to undergo phase variation to decrease or produce modified forms of the O1 antigen to evade ICP1 infection (*7*). However, the fact that resistant strains gave a positive serologic response when tested for the O1 antigen suggests that there are mechanisms for resistance to ICP1 that lie elsewhere in the genome.

SXT-ICE has been reported to have 5 hotspots within the accessory region, including hotspot 5 (*Vch*Ind5), which confers resistance to ICP1 phage infection (*12*). When we evaluated the SXT-ICE sequence in all 24 DRC *V. cholerae* genomes, we found that all harbored genes identical to the wild-type SXT-ICE, which should make the strains phage-resistant. However, as already noted, the DRC ICP1 phage that we identified encodes the anti-BREX factor OrbA, which protects against the host *Vch*Ind5 (*12*). We did not detect other mechanisms usually associated with resistance of host cells to phage, such as acquisition and expression of a family of phage-inducible chromosomal island–like elements (*17*), and none of the DRC ICP1 isolates encoded a previously described CRISPR-cas system specifically targeting phage-inducible chromosomal island–like elements for destruction, which might enable the phage to evade host immunity (*29*). At this point we cannot comment further on the mechanisms underlying resistance of our DRC *V. cholerae* strains to the regional DRC ICP1 phage other than to note the complexity of these regional phage/host interactions.

**Figure 3:**
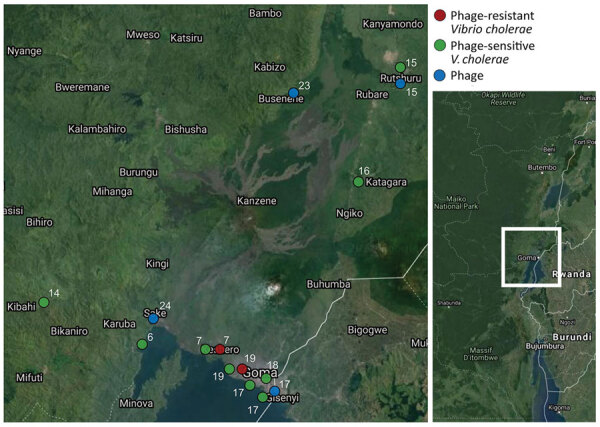
Sampling locations of phages and phage-resistant or sensitive *Vibrio cholerae* isolates in the Democratic Republic of the Congo. Each sampling location is coded by color (key) and number, which also appear at the tips of the maximum clade credibility tree in Figure 4 for comparison. Inset shows location of sampling area in the Democratic Republic of the Congo.

Comparison of genome-wide weighted averages of *dS* and *dN* along the internal branches of the cholera phylogeny showed a *dN*/*dS* ratio significantly >1 (p<0.001) (Appendix Figure 3, panel A). Moreover, the difference between *dN* and *dS* divergence accumulating over time along the internal branches of the phylogeny also appears to be increasing (Appendix Figure 3, panel B). In other words, the mixed presence of susceptible and resistant *V. cholerae* phenotypes, at least in the DRC Goma region where samples were collected, together with *dN*/*dS* patterns suggest that *V. cholerae* has been evolving under pressure of increasing diversifying selection, possibly driven by the co-circulation of predatory phages. Indeed, the map of sampling locations shows that phage-resistant or phage-susceptible *V. cholerae* strains, as well as independently sampled phages, have tended to co-circulate in the DRC Goma region and surrounding locales (Figure 3).

**Figure 4:**
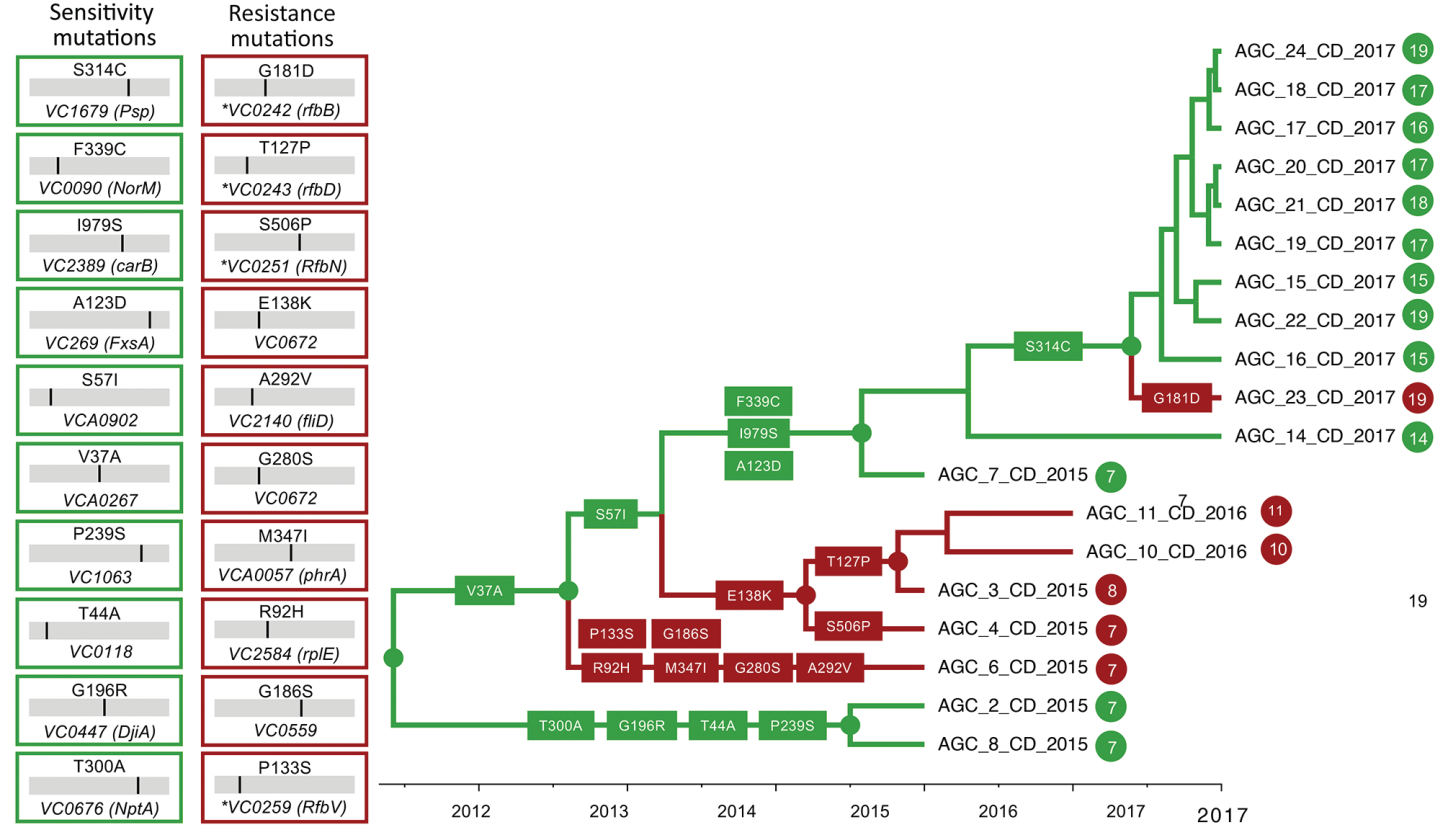
Phage-resistant or phase-sensitive dynamics and mutational patterns of *Vibrio cholerae* isolates in the Democratic Republic of the Congo. The boxes show the mutations that have been found along the backbone, internal, or external branches of the maximum clade credibility tree. Each number is the amino acid position of the protein where the mutation was mapped in the tree. The branches are scaled in time and colored on the basis of resistance (red) or sensitivity (green), matching colors in Figure 3. Circles and colors in internal node indicate posterior probability support >0.9 for an ancestor to be resistant or sensitive.

To examine in more detail the adaptive fitness landscape that might confer either resistance or sensitivity to phage predation, we optimized an MCC tree for the subset of *V. cholerae* sequences including all strains in the ϕ_R/S_ clade, as well as 2 outgroup strains, AGC-2-CD-2015 and AGC-8-CD-2015, that clustered outside the clade (Figure 4). We used a Bayesian phylogeographic model with phage resistance or susceptibility as discrete phenotypic characters to infer the most likely phenotype of the ancestral (internal) nodes of the tree. The analysis clearly shows that the backbone path (trunk) of the ϕ_R/S_ clade, which represents the surviving lineage successfully propagating through time (*28*), is dominated by isolates with the phage-sensitive phenotype and connects phage-sensitive ancestral sequences that first generated a subcluster of strains circulating in 2015–2016 and then a subcluster including 2017 strains.

We cannot say which mutations are responsible for acquisition of phage resistance, but mutations in genes belonging to the O1-antigen biosynthetic gene cluster appear to have emerged, independently, along 3 distinct evolutionary lineages. The first lineage, leading to strain AGC-6-2015-DRC sampled in 2015, is characterized by amino acid substitutions in the *rfbV*, *rpIE*, *phrA*, and *fliD* genes. The second lineage resulted in a monophyletic clade of phage-resistant strains with mutations in either *rfbN* (strains sampled in 2015) or *rfbD* (sampled in 2016). The third lineage, leading to strain AGC-23-2017-DRC (sampled in 2017), was characterized again by an amino acid substitution in the *rfbB* gene.

## Discussion

Cholera continues to be a major public health problem in the Great Lakes region of Africa (*1*–*4*). To optimize cholera control and appropriately target public health interventions, evolutionary drivers for *V. cholerae* in this area need to be determined, including reasons why certain *V. cholerae* strains emerge and persist while others fail to propagate. Our data provide further information on sources and subsequent development of endemic *V. cholerae* O1 in eastern DRC. Our work also highlights the effects a novel regional bacteriophage can have on cholera evolution. As reflected in the trunk of the phylogeny of the ϕ_R/S_ clade, *V. cholerae* isolates displaying the phage-sensitive phenotype appear to be successfully propagating, with every branch that leads to phage-resistant phenotypes in the phylogeny eventually dying out. Our findings are somewhat counterintuitive: phage resistance, rather than encouraging expansion of the epidemic clone, led to evolutionary dead ends; however, our data highlight the ability of *V. cholerae* to explore and quickly abandon different evolutionary pathways during epidemic spread. This finding is not surprising considering the potential fitness cost of phage resistance, particularly if resistance results in mutants highly attenuated for virulence (*30*,*31*). Further work will be needed to determine the exact mechanism by which the *V. cholerae* strains isolated in this study were either completely or partially resistant (turbid plaque) to ICP1_2017_A_DRC and whether the phage can mutate to regain virulence (*12*).

In summary, our study documents a complex co-evolutionary dynamic involving *V. cholerae* and predatory phages (*30*) in the Great Lakes region of DRC. Phage-sensitive and highly infectious strains co-circulate with phage-resistant ones that occasionally emerge and eventually die out along different evolutionary pathways in response to the presence or absence of predatory phages in the environment, although the main phage-sensitive evolutionary lineage continued to propagate over time. The ability of *V. cholerae* to explore multiple mutational pathways in different genes and achieve phage resistance provides a substantial evolutionary advantage in terms of quick adaptive response to a changing environment, leading to emergence of new strains. Continuous monitoring of toxigenic *V. cholerae* and predator ICP1 phages in both patient fecal samples and aquatic environments in DRC and elsewhere could provide invaluable epidemiologic data for monitoring the spread of cholera, identifying environmental actors driving successful dissemination, and assessing the potential for new outbreaks.

Appendix Additional information on emergence and evolutionary response of *Vibrio cholerae* to a novel bacteriophage in the Democratic Republic of the Congo.
