# Supplementary material for: Emergence and Evolutionary Response of Vibrio cholerae to Novel Bacteriophage, Democratic Republic of the Congo
Source: Emerg Infect Dis. 2022 Dec;28(12):2482–90. doi: 10.3201/eid2812.220572 (PMC9707599; doi:10.3201/eid2812.220572)
Supplement: Appendix — Additional information on emergence and evolutionary response of Vibrio cholerae to a novel bacteriophage in the Democratic Republic of the Congo. [file 22-0572-Techapp-s1.pdf]

Article DOI: https://doi.org/10.3201/eid2812.220572

Emergence and Evolutionary Response of *Vibrio cholerae* to a Novel Bacteriophage in the Democratic Republic of the Congo

Appendix

Materials and Methods

Isolation and Characterization of Toxigenic *Vibrio cholerae* O1 and Virulent Phages

During 2015–2017, fecal samples from suspected cholera patients admitted in different cholera treatment centers around Goma, Democratic Republic of the Congo (DRC) (Table) were placed on Cary-Blair transport media and brought to the Laboratoire Provincial de Sante Publique du Nord-Kivu in Goma for microbiological and serologic analysis. *V. cholerae* in fecal samples were enriched in alkaline peptone water as described elsewhere (*1*). Following enrichment, a loopful of culture was streaked onto thiosulfate citrate bile salts (TCBS) agar and the plates were incubated overnight at 37°C. Bacterial colonies grown as yellow color on TCBS agar were subcultured onto Luria-Bertani, Miller (LB) agar and the culture plates were incubated overnight at 37°C. To determine serogroup, translucent colonies grown on LB-agar were tested against polyvalent antiserum specific for *V. cholerae* O1 and O139 by slide agglutination tests; each O1 positive strain was further typed for serotype using antiserum specific for Ogawa, Inaba, or Hikojima serotype. The toxigenic *V. cholerae* were stored in soft LB-agar (0.7% agar) and sent to the Emerging Pathogens Institute (EPI) at University of Florida for further analysis.

For isolation of potential virulent phages, cholera rice-water fecal samples collected between 2016 and 2017 were centrifuged at 5,000 x g for 10 min in a microfuge. Fecal samples sused to detect and characterize virulent phages were different than the fecal samples used for detection of 24 *V. cholerae* O1 strains described above. The resultant supernatant was filtered through a 0.22 µm syringe filter, stored at 4°C in a sterile microfuge tube, and sent to EPI for further analysis. For further characterization of potential phages in cholera-confirmed patients' fecal samples, 41 fecal sample filtrates were brought to EPI.

Virulent phage plaque assay: For detection and characterization of potential virulent phages, each filtered fecal sample was tested by plaque assay using a host toxigenic *V. cholera*e O1 Inaba strain, AGC-15 (Appendix Table 1). Isolated in DRC, AGC-15 has a wild-type *ompU* sequence, which encodes the receptor for ICP2. AGC-15 also has the O1-antigen receptor for ICP1 and ICP3 (*2*) and lacks any PLE elements mediating immunity to ICP1. Briefly, a sterile glass tube was inoculated with 100 µl of filtered-sterilized fecal sample (potential source of virulent phage), 9.8 ml of LB-broth, and 100 µl of host *V. cholerae* AGC-15 culture (freshly grown to mid-exponential phase). The culture mixture was incubated overnight at 37°C with aeration to enrich any phages capable of infecting AGC-15. Following incubation, the culture was transferred to a 15 ml conical tube and centrifuged for 10 min at 5,000 x g at 4°C. To eliminate residual bacterial cells, the supernatant was filtered through a 0.22 µm syringe filter and the filtrate was stored at 4°C. The filtered supernatant was serially diluted (10-fold) in LB-broth to reach a dilution of 10^{-5} in a 96-well microtiter plate. One hundred µl of AGC-15 culture (grown to a mid-exponential phase) were added to the undiluted and each serially diluted filtrate in the microtiter plate and was incubated at room temperature for 10 min to enable phage adsorption. All 190 µl of each mixture from the microtiter plate was transferred to wells of six-well tissue culture plates.

Three ml of soft LB-agar (0.35% agar) kept at 55°C in a water bath were added to each well of the six-well plate and the plate was gently swirled to mix the bacteria and phage evenly. To allow the agar to solidify, the plate was left for 30 min at room temperature followed by incubation at 37°C for 3–4 hours. After incubation, the plate was visually observed for plaque formation. If no plaques were observed after 4 hours of incubation, the plate was incubated overnight at room temperature to determine if any plaques were formed following extended incubation time. For virulent phage purification, a single clear plaque was picked using a Pasteur pipette into 1 ml of LB-broth and incubated overnight at 4°C to allow phage to diffuse out of the soft agar piece. High titer stocks of purified plaques were made by multiplication in broth culture as described above.

Whole genome mapping and hqSNP calling: Toxigenic *V. cholerae* O1 samples collected were confirmed by serology and PCR (*1*). After subculture, gDNA extraction was performed using the Qiagen DNeasy Blood and Tissue kit. Genomic DNA from all isolates were cultured and extracted from a bacterial pellet. Sample library construction was performed using the

Nextera XT DNA Library Preparation Kit (Illumina, https://www.illumina.com). Whole-genome sequencing on all isolates was performed with the Illumina MiSeq for 500 cycles. Adaptor and raw sequence reads were filtered by length and quality by using the program Trimmomatic (*3*). After quality filtering, Bowtie2 (*4*) was used to map the sequence reads to the reference genome, *V. cholerae* O1 str. N16961 (GenBank Accessions: NC_002505.1 and NC_002506.1) (*5*). After reads were mapped to the reference genome, duplicate reads were marked and realigned using Picard (http://broadinstitute.github.io/picard). The reference-based mapping alignment was then verified and fixed accordingly. Freebayes (Garrison MG, unpub data; https://arxiv.org/abs/1207.3907) was used to create a custom genome-wide SNP calling database (dbSNP) from all isolates in the dataset to perform base quality score recalibration (BQSR), as outlined in GATK's best practice guidelines for germline variation (https://software.broadinstitute.org/gatk/). The newly created variant call format (VCF) file obtained from Freebayes was subsequently filtered only for SNPs. The VCF file was used as a dbSNP for BQSR of the reference-based mapped alignment files; alignment files were then recalibrated and variants calling on the newly recalibrated files performed with Freebayes. The newly created VCF file was filtered only for SNPs and normalized using the program BCFtools (http://www.htslib.org/doc/bcftools.html). Normalization simplifies the represented variants in the VCF file by showing as few bases as possible at particular SNP sites in the genome. SNPs were filtered by depth of coverage, quality, and genotype likelihood, as described in Azarian *et al.* (*6*). Finally, the SNP FASTA alignment was extracted by a custom python script from the VCF file. The SNP alignment was filtered site-by-site, leaving sites with only greater than 75% of SNPs at that particular site, making a high-quality SNP (hqSNP) alignment. Our hqSNP alignment was then annotated using the program SnpEff (*7*). The final genome-wide SNP alignment included 120 T10 sublineage *V. cholerae* genome sequences (Appendix Table 1): 24 strains were collected in DRC (eight collected in 2015, five in 2016, 11 in 2017) and sequenced in this study; 71 were publicly available genomes from outbreaks in eastern DRC between 2014 and 2016 (*8*); six archival and publicly available DRC genomes collected between 2001–2013; 17 genomes collected across Africa between 1998 and 2014; and two publicly available genomes from India, ancestor of T10 sublineage (*9*). While the previously available DRC samples spanned 2014–2016 (*10*), we expanded the temporal dimension of the DRC collection as we sequenced mainly strains collected in 2017. The MLST analysis was performed on the online

tool PubMLST (Jolley K, unpub data; https://doi.org/10.12688/wellcomeopenres.14826.1); results are shown in Appendix Table 2.

Phylogenetic and temporal signal: All datasets used in this study passed phylogenetic quality checks such as evaluating the presence of phylogenetic signal, to resolve the phylogenetic relationship among the *V. cholerae* isolates, and temporal signal for a robust calibration of the molecular clock (Appendix Figure 1). We performed likelihood mapping analysis using IQ-TREE (*10*), which enables the report likelihood values of the three possible unrooted trees, inferred using the best-fitting nucleotide substitution model, of each possible quartet (set of four sequences) on an equilateral triangle (likelihood map). In a likelihood map, dots (likelihood values) in the center of the triangle represent phylogenetic noise and simulation have shown that datasets with <35% nose (as it was in our case) can reliably be used for phylogeny inference (*11*). For each dataset, the presence of temporal signal was assessed by calculating the tree root-to-tip divergence regression plot with TempEst v1.5 (http://tree.bio.ed.ac.uk/software/tempest) (*12*), using maximum-likelihood (ML) phylogenies inferred with IQ-TREE (*10*) and the best-fitting nucleotide substitution model according to Bayesian Information Criterion (BIC), and ultrafast bootstrap (BB) approximation (1000 replicates) to assess robustness of the phylogeny internal branches.

Bayesian Phylogeography of DRC isolates: To test the hypothesis of whether cholera outbreaks in the DRC were caused by endemic *V. cholerae* O1 strains, or strains recently introduced from other African countries surrounding the Great Lakes region, we used the Bayesian phylogeographic (*13*) coalescent-based method (*14*) implemented in the BEAST v1.10.4 (*15*) software package. The reconstruction of *V. cholerae* O1 spatiotemporal spread from different locations through Bayesian phylogeography requires the calibration of a molecular clock. Evolutionary rates were estimated implementing a HKY nucleotide substitution model (*16*) with empirical base frequencies, gamma distribution of site-specific rate heterogeneity, and ascertainment bias correction (*17*), testing a constant demographic prior against non-parametric demographic models – Gaussian Markov randomfield Skyride (BSR) (*18*) and Bayesian Skyline Plot (BSP) (*19*) – to rule out spurious changes in effective population size inferred by a non-parametric model that would in turn effect timing of divergence events (*20*). Additionally, for each demographic model, we compared a strict and relaxed uncorrelated (lognormal distribution among branches) molecular clock (*21*). The best fitting molecular clock and demographic model

were chosen by estimating the marginal likelihood of each model by using path sampling (PS) and stepping-stone (SS) methods, followed by Bayes Factor comparison test (*11*,*15*). A Markov Chain Monte Carlo (MCMC) sampler was run for 500 million generations, sampling every 50,000 generations. Proper mixing of the Markov chain was evaluated by the effective population size (ESS) of each parameter estimate under a specific model. ESS values >200 for all parameter estimates are considered as evidence of proper mixing in the analysis. The sampling location for each isolate was used as a discreet trait to reconstruct likely locations of ancestral sequences (internal nodes in the tree) and infer migration events (bacterial flow) that took place in the DRC and throughout the Great Lakes Region. Phylogeographic analysis was performed with the BEAST package v1.10.4 (*15*). Transitions between discrete states (location of where the isolate was collected) were estimated using the continuous-time Markov chain model operating the asymmetric migration model with Bayesian Stochastic Search Variable Selection (*13*). In our reconstruction of ancestral states, we assume migration occurs along branches connecting the tree nodes. The maximum clade credibility (MCC) tree chosen from the posterior distribution of trees using TreeAnnotator v1.10.4 after 10% burn-in. The MCC tree was annotated in R using the package ggtree (*22*) for publishing purposes.

Calculation of weighted average of nonsynonmous (*dN*) and synonymous substitution rates (*dS*), and selection analysis: A codon alignment from the DRC clade was generated to analyze other mutations in the *V. cholerae* genome from the isolates in the phylogeny, and a subset of 200 Bayesian MCC genealogies randomly was obtained from the posterior distribution of trees for each subsampled dataset. The weighted average of synonymous substitution rates (*dS*) and non-synonymous substitution rates (*dN*) in the protein-coding regions of the *V. cholerae* O1 genome for all, internal and external branches were obtained from a subset of 200 Bayesian MCC trees randomly obtained from the posterior distribution of trees, as described by Lemey et al. (*23*). The subset of trees and in-house java scripts were then used to calculate *dN/dS* rates and divergence in the isolates located within the DRC clade and plotted using the package ggplot2 in R.

Whole genome sequencing, genome assembly and annotation of DRC phages

To characterize DRC phages, we plaque-purified phages from eight different patient samples, and prepared high titer stocks of each of these phages. Sample library construction for whole-genome sequencing was performed using the Nextera XT DNA Library Preparation Kit

(Illumina). Whole-genome sequencing was performed with the Illumina MiSeq for 50 cycles. We obtained over 200-fold coverage facilitating de novo assembly of each phage genome into one complete contig using CLC Genomics Workbench (QIAGEN, https://www.qiagen.com). Manual confirmation/correction of low coverage areas and/or problem areas were performed to ensure authentic genome assembly. Annotation of phage genomes was performed as described elsewhere (*24*). Briefly, open reading frames from existing annotated ICP1 phages were compared against the DRC phage genomes using BLASTn to find homologs. Additional new putative open reading frames were discovered with de novo prediction software and added to the annotation. This was performed for each of the eight phages and due to very high similarity between them, one representative was randomly chosen and designated as ICP1_2017_A_DRC. This representative was aligned with existing ICP1 phage genomes using Mauve (*25*) and a maximum-likelihood, bootstrapped phylogenetic tree was generated with PhyML (-s BEST 92–rand_start–n_rand_starts 10 -b 100) (*26*).

References

1. Ali A, Chen Y, Johnson JA, Redden E, Mayette Y, Rashid MH, et al. Recent clonal origin of cholera in Haiti. Emerg Infect Dis. 2011;17:699–701. PubMed https://doi.org/10.3201/eid1704.101973

2. Seed KD, Bodi KL, Kropinski AM, Ackermann HW, Calderwood SB, Qadri F, et al. Evidence of a dominant lineage of *Vibrio cholerae*-specific lytic bacteriophages shed by cholera patients over a 10-year period in Dhaka, Bangladesh. MBio. 2011;2:e00334–10. PubMed https://doi.org/10.1128/mBio.00334-10

3. Bolger AM, Lohse M, Usadel B. Trimmomatic: a flexible trimmer for Illumina sequence data. Bioinformatics. 2014;30:2114–20. PubMed https://doi.org/10.1093/bioinformatics/btu170

4. Langmead B, Salzberg SL. Fast gapped-read alignment with Bowtie 2. Nat Methods. 2012;9:357–9. PubMed https://doi.org/10.1038/nmeth.1923

5. Heidelberg JF, Eisen JA, Nelson WC, Clayton RA, Gwinn ML, Dodson RJ, et al. DNA sequence of both chromosomes of the cholera pathogen *Vibrio cholerae.* Nature. 2000;406:477–83. PubMed https://doi.org/10.1038/35020000

6. Azarian T, Ali A, Johnson JA, Mohr D, Prosperi M, Veras NM, et al. Phylodynamic analysis of clinical and environmental *Vibrio cholerae* isolates from Haiti reveals diversification driven by positive selection. MBio. 2014;5:5. PubMed https://doi.org/10.1128/mBio.01824-14

7. Cingolani P, Platts A, Wang L, Coon M, Nguyen T, Wang L, et al. A program for annotating and predicting the effects of single nucleotide polymorphisms, SnpEff: SNPs in the genome of *Drosophila melanogaster* strain w1118; iso-2; iso-3. Fly (Austin). 2012;6:80–92. PubMed https://doi.org/10.4161/fly.19695

8. Irenge LM, Ambroise J, Mitangala PN, Bearzatto B, Kabangwa RKS, Durant JF, et al. Genomic analysis of pathogenic isolates of Vibrio cholerae from eastern Democratic Republic of the Congo (2014-2017). PLoS Negl Trop Dis. 2020;14:e0007642. PubMed https://doi.org/10.1371/journal.pntd.0007642

9. Weill FX, Domman D, Njamkepo E, Tarr C, Rauzier J, Fawal N, et al. Genomic history of the seventh pandemic of cholera in Africa. Science. 2017;358:785–9. PubMed https://doi.org/10.1126/science.aad5901

10. Nguyen LT, Schmidt HA, von Haeseler A, Minh BQ. IQ-TREE: a fast and effective stochastic algorithm for estimating maximum-likelihood phylogenies. Mol Biol Evol. 2015;32:268–74. PubMed https://doi.org/10.1093/molbev/msu300

11. Schmidt HA, Strimmer K, Vingron M, von Haeseler A. TREE-PUZZLE: maximum likelihood phylogenetic analysis using quartets and parallel computing. Bioinformatics. 2002;18:502–4. PubMed https://doi.org/10.1093/bioinformatics/18.3.502

12. Rambaut A, Lam TT, Max Carvalho L, Pybus OG. Exploring the temporal structure of heterochronous sequences using TempEst (formerly Path-O-Gen). Virus Evol. 2016;2:vew007. PubMed https://doi.org/10.1093/ve/vew007

13. Lemey P, Rambaut A, Drummond AJ, Suchard MA. Bayesian phylogeography finds its roots. PLOS Comput Biol. 2009;5:e1000520. PubMed https://doi.org/10.1371/journal.pcbi.1000520

14. Grenfell BT, Pybus OG, Gog JR, Wood JL, Daly JM, Mumford JA, et al. Unifying the epidemiological and evolutionary dynamics of pathogens. Science. 2004;303:327–32. PubMed https://doi.org/10.1126/science.1090727

15. Drummond AJ, Rambaut A. BEAST: Bayesian evolutionary analysis by sampling trees. BMC Evol Biol. 2007;7:214. PubMed https://doi.org/10.1186/1471-2148-7-214

16. Hasegawa M, Kishino H, Yano T. Dating of the human-ape splitting by a molecular clock of mitochondrial DNA. J Mol Evol. 1985;22:160–74. PubMed https://doi.org/10.1007/BF02101694

17. Leaché AD, Banbury BL, Felsenstein J, de Oca AN, Stamatakis A. Short tree, long tree, right tree, wrong tree: new acquisition bias corrections for inferring SNP phylogenies. Syst Biol. 2015;64:1032–47. PubMed https://doi.org/10.1093/sysbio/syv053

18. Minin VN, Bloomquist EW, Suchard MA. Smooth skyride through a rough skyline: Bayesian coalescent-based inference of population dynamics. Mol Biol Evol. 2008;25:1459–71. PubMed https://doi.org/10.1093/molbev/msn090

19. Strimmer K, Pybus OG. Exploring the demographic history of DNA sequences using the generalized skyline plot. Mol Biol Evol. 2001;18:2298–305. PubMed https://doi.org/10.1093/oxfordjournals.molbev.a003776

20. Hall MD, Woolhouse ME, Rambaut A. The effects of sampling strategy on the quality of reconstruction of viral population dynamics using Bayesian skyline family coalescent methods: A simulation study. Virus Evol. 2016;2:vew003. PubMed https://doi.org/10.1093/ve/vew003

21. Drummond AJ, Rambaut A, Shapiro B, Pybus OG. Bayesian coalescent inference of past population dynamics from molecular sequences. Mol Biol Evol. 2005;22:1185–92. PubMed https://doi.org/10.1093/molbev/msi103

22. Yu GC, Smith DK, Zhu HC, Guan Y, Lam TTY. GGTREE: an R package for visualization and annotation of phylogenetic trees with their covariates and other associated data. Methods Ecol Evol. 2017;8:28–36. https://doi.org/10.1111/2041-210X.12628

23. Lemey P, Kosakovsky Pond SL, Drummond AJ, Pybus OG, Shapiro B, Barroso H, et al. Synonymous substitution rates predict HIV disease progression as a result of underlying replication dynamics. PLOS Comput Biol. 2007;3:e29. PubMed https://doi.org/10.1371/journal.pcbi.0030029

24. Angermeyer A, Das MM, Singh DV, Seed KD. Analysis of 19 highly conserved *Vibrio cholerae* bacteriophages isolated from environmental and patient sources over a twelve-year period. Viruses. 2018;10:10. PubMed https://doi.org/10.3390/v10060299

25. Darling AE, Mau B, Perna NT. progressiveMauve: multiple genome alignment with gene gain, loss and rearrangement. PLoS One. 2010;5:e11147. PubMed https://doi.org/10.1371/journal.pone.0011147

26. Guindon S, Dufayard JF, Lefort V, Anisimova M, Hordijk W, Gascuel O. New algorithms and methods to estimate maximum-likelihood phylogenies: assessing the performance of PhyML 3.0. Syst Biol. 2010;59:307–21. PubMed https://doi.org/10.1093/sysbio/syq010

Appendix Table 1. *Vibrio cholera* strains included in phylogenetic studies in Democratic Republic of the Congo*

V. cholera name	Year	Location, country or sea	Location, region/ locality	Lineage	Latitude	Longitude	Location on map†	SRA ID
AGC_1_CD_2015	2015	DRC	North Kivu/ Kirotshe	ST515	−1.613051	29.03132	6	SRR15192533
AGC_2_CD_2015	2015	DRC	Goma/ Buhimba	ST69	−1.621214	29.156623	7	SRR15192532
AGC_3_CD_2015	2015	DRC	Mutwanga	ST515	0.514939	25.191932	8	SRR15192521
AGC_4_CD_2015	2015	DRC	Goma/ Buhimba	ST515	−1.621214	29.156623	7	SRR15192516
AGC_5_CD_2015	2015	DRC	Goma/ Buhimba	ST515	−1.621214	29.156623	7	SRR15192515
AGC_6_CD_2015	2015	DRC	Goma/ Buhimba	ST515	−1.621214	29.156623	7	SRR15192514
AGC_7_CD_2015	2015	DRC	Goma/ Buhimba	ST515	−1.621214	29.156623	7	SRR15192513
AGC_8_CD_2015	2015	DRC	Goma/ Buhimba	ST515	−1.621214	29.156623	7	SRR15192512
AGC_9_CD_2016	2016	DRC	Maniema/ Kabambare	ST515	−4.400901	27.765835	9	SRR15192511
AGC_10_CD_2016	2016	DRC	Karisimbi/ Hop Millitaire	ST515	1.5064	29.4508	10	SRR15192510
AGC_11_CD_2016	2016	DRC	Alimbongo	ST515	−0.36879	29.156179	11	SRR15192531
AGC_12_CD_2016	2016	DRC	South Kivu/ Fizi	ST515	−4.30058	28.94212	12	SRR15192530
AGC_13_CD_2016	2016	DRC	South Kivu/ Kimbilulenge	ST515	−3.21838	28.25855	13	SRR15192529
AGC_14_CD_2017	2017	DRC	Kirotshe/ Rubaya	ST515	−1.546277	28.873122	14	SRR15192528
AGC_15_CD_2017	2017	DRC	Rutshuru/ Hgr	ST515	−1.188054595	29.4459123	15	SRR15192527
AGC_16_CD_2017	2017	DRC	Rutshuru/ Hgr	ST515	−1.188054595	29.4459123	15	SRR15192526
AGC_17_CD_2017	2017	DRC	Nyiragongo/ Turunga	ST515	−1.3527161	29.37873	16	SRR15192525
AGC_18_CD_2017	2017	DRC	Goma/Hop Provincial	ST515	−1.678865426	29.8	17	SRR15192524
AGC_19_CD_2017	2017	DRC	Goma/Hop Provincial	ST515	−1.678865426	29.8	17	SRR15192523
AGC_20_CD_2017	2017	DRC	Goma/Hop Provincial	ST515	−1.678865426	29.8	17	SRR15192522
AGC_21_CD_2017	2017	DRC	Karisimbi/ Prison centrale	ST69	−1.9	29	18	SRR15192520
AGC_22_CD_2017	2017	DRC	Karisimbi/ Majengo	ST69	−1.65388	29.5	19	SRR15192519
AGC_23_CD_2017	2017	DRC	Karisimbi/ Majengo	ST515	−1.65388	29.5	19	SRR15192518
AGC_24_CD_2017	2017	DRC	Karisimbi/ Majengo	ST515	−1.65388	29.5	19	SRR15192517
ERR019292_KE_2007	2007	Kenya						ERR019292
ERR037738_KE_2010	2010	Kenya						ERR037738
ERR044795_ZM_2003	2003	Zambia						ERR044795
ERR1877642_RW_2000	2000	Rwanda						ERR1877642
ERR1878097_CD_2003	2003	DRC						ERR1878097
ERR1878101_CD_2002	2002	DRC	Congo/ Zaire		−11.64112	27.51818	3	ERR1878101
ERR1878103_KM_2003	2003	Comoros						ERR1878103
ERR1878154_KE_2006	2006	Kenya						ERR1878154
ERR1878551_DJ_2007	2007	Djibouti						ERR1878551
ERR1879386_TZ_1998	1998	Tanzania						ERR1879386
ERR1879540_KE_1998	1998	Kenya						ERR1879540

V. cholera name	Year	Location, country or sea	Location, region/ locality	Lineage	Latitude	Longitude	Location on map†	SRA ID
ERR1880767_TZ_1998	1998	Tanzania						ERR1880767
ERR1880801_IN_1997	1997	India						ERR1880801
ERR1880812_IN_1998	1998	India						ERR1880812
ERR2265670_NE_2014	2014	Niger						ERR2265670
ERR3268992_CD_2017	2017	DRC	Minova		−4.32153	15.31185	3	ERR3268992
ERR3268993_CD_2015	2015	DRC	Goma		−1.6835	29.2356	17	ERR3268993
ERR3268994_CD_2015	2015	DRC	Goma		−1.6835	29.2356	17	ERR3268994
ERR3268995_CD_2015	2015	DRC	Goma		−1.6835	29.2356	17	ERR3268995
ERR3268996_CD_2015	2015	DRC	Goma		−1.6835	29.2356	17	ERR3268996
ERR3268997_CD_2015	2015	DRC	Goma		−1.6835	29.2356	17	ERR3268997
ERR3268998_CD_2015	2015	DRC	Goma		−1.6835	29.2356	17	ERR3268998
ERR3268999_CD_2015	2015	DRC	Goma		−1.6835	29.2356	17	ERR3268999
ERR3269000_CD_2015	2015	DRC	Goma		−1.6835	29.2356	17	ERR3269000
ERR3269001_CD_2015	2015	DRC	Goma		−1.6835	29.2356	17	ERR3269001
ERR3269002_CD_2015	2015	DRC	Goma		−1.6835	29.2356	17	ERR3269002
ERR3269003_CD_2015	2015	DRC	Goma		−1.6835	29.2356	17	ERR3269003
ERR3269004_CD_2015	2015	DRC	Goma		−1.6835	29.2356	17	ERR3269004
ERR3269005_CD_2015	2015	DRC	Goma		−1.6835	29.2356	17	ERR3269005
ERR3269006_CD_2015	2015	DRC	Goma	ST515	−1.6835	29.2356	17	ERR3269006
ERR3269007_CD_2015	2015	DRC	Fizi-Baraka	ST515	−4.30058	28.94212	12	ERR3269007
ERR3269008_CD_2015	2015	DRC	Fizi-Baraka	ST515	−4.30058	28.94212	12	ERR3269008
ERR3269009_CD_2015	2015	DRC	Fizi-Baraka	ST515	−4.30058	28.94212	12	ERR3269009
ERR3269010_CD_2015	2015	DRC	Fizi-Baraka	ST515	−4.30058	28.94212	12	ERR3269010
ERR3269011_CD_2015	2015	DRC	Bukavu	ST515	−2.50316	28.85309	21	ERR3269011
ERR3269012_CD_2015	2015	DRC	Fizi-Baraka	ST515	−4.30058	28.94212	12	ERR3269012
ERR3269013_CD_2015	2015	DRC	Minova	ST515	−4.32153	15.31185	3	ERR3269013
ERR3269014_CD_2016	2016	DRC	Fizi-Baraka	ST515	−4.30058	28.94212	12	ERR3269014
ERR3269015_CD_2016	2016	DRC	Fizi-Baraka	ST515	−4.30058	28.94212	12	ERR3269015
ERR3269016_CD_2016	2016	DRC	Fizi-Baraka	ST515	−4.30058	28.94212	12	ERR3269016
ERR3269017_CD_2016	2016	DRC	Fizi-Baraka	ST515	−4.30058	28.94212	12	ERR3269017
ERR3269018_CD_2016	2016	DRC	Fizi-Baraka	ST515	−4.30058	28.94212	12	ERR3269018
ERR3269019_CD_2016	2016	DRC	Fizi-Baraka	ST515	−4.30058	28.94212	12	ERR3269019
ERR3269020_CD_2016	2016	DRC	Uvira	ST515	−3.38413	29.1415	20	ERR3269020
ERR3269021_CD_2016	2016	DRC	Uvira	ST515	−3.38413	29.1415	20	ERR3269021

V. cholera name	Year	Location, country or sea	Location, region/ locality	Lineage	Latitude	Longitude	Location on map†	SRA ID
ERR3269022_CD_2016	2016	DRC	Uvira	ST69	−3.38413	29.1415	20	ERR3269022
ERR3269023_CD_2016	2016	DRC	Uvira	ST69	−3.38413	29.1415	20	ERR3269023
ERR3269024_CD_2016	2016	DRC	Uvira	ST69	−3.38413	29.1415	20	ERR3269024
ERR3269025_CD_2016	2016	DRC	Uvira	ST69	−3.38413	29.1415	20	ERR3269025
ERR3269026_CD_2016	2016	DRC	Uvira	ST515	−3.38413	29.1415	20	ERR3269026
ERR3269027_CD_2016	2016	DRC	Fizi-Baraka	ST69	−4.30058	28.94212	12	ERR3269027
ERR3269028_CD_2015	2015	DRC	Fizi-Baraka	ST515	−4.30058	28.94212	12	ERR3269028
ERR3269029_CD_2015	2015	DRC	Fizi-Baraka	ST515	−4.30058	28.94212	12	ERR3269029
ERR3269031_CD_2016	2016	DRC	Alimbongo	ST515	−0.3692	29.155569	8	ERR3269031
ERR3269033_CD_2016	2016	DRC	Kabambare	ST69	−4.68967	27.69298	9	ERR3269033
ERR3269034_CD_2016	2016	DRC	Fizi-Baraka	ST69	−4.30058	28.94212	12	ERR3269034
ERR3269035_CD_2016	2016	DRC	Fizi-Baraka	ST515	−4.30058	28.94212	12	ERR3269035
ERR3269036_CD_2016	2016	DRC	Fizi-Baraka	ST69	−4.30058	28.94212	12	ERR3269036
ERR3269037_CD_2016	2016	DRC	Kimbilulenge	ST69	−4.32153	15.31185	3	ERR3269037
ERR3269038_CD_2016	2016	DRC	Kimbilulenge	ST69	−4.32153	15.31185	3	ERR3269038
ERR3269039_CD_2014	2014	DRC	Fizi-Baraka	ST69	−4.30058	28.94212	12	ERR3269039
ERR3269040_CD_2014	2014	DRC	Fizi-Baraka	ST69	−4.30058	28.94212	12	ERR3269040
ERR3269041_CD_2014	2014	DRC	Fizi-Baraka	ST69	−4.30058	28.94212	12	ERR3269041
ERR3269042_CD_2014	2014	DRC	Alimbongo	ST69	−0.3692	29.155569	8	ERR3269042
ERR3269043_CD_2015	2015	DRC	Masisi	ST69	−1.3527161	29.37873	16	ERR3269043
ERR3269044_CD_2014	2014	DRC	Fizi-Baraka	ST69	−4.30058	28.94212	12	ERR3269044
ERR3269045_CD_2014	2014	DRC	Fizi-Baraka	ST69	−4.30058	28.94212	12	ERR3269045
ERR3269047_CD_2015	2015	DRC	Goma	ST69	−1.6835	29.2356	17	ERR3269047
ERR3269048_CD_2015	2015	DRC	Goma	ST515	−1.6835	29.2356	17	ERR3269048
ERR3269049_CD_2015	2015	DRC	Goma	ST69	−1.6835	29.2356	17	ERR3269049
ERR3269050_CD_2015	2015	DRC	Goma	ST69	−1.6835	29.2356	17	ERR3269050
ERR3269051_CD_2014	2014	DRC	Uvira	ST69	−3.38413	29.1415	20	ERR3269051
ERR3269052_CD_2014	2014	DRC	Uvira	ST69	−3.38413	29.1415	20	ERR3269052
ERR3269053_CD_2014	2014	DRC	Uvira	ST69	−3.38413	29.1415	20	ERR3269053
ERR3269054_CD_2014	2014	DRC	Uvira	ST69	−3.38413	29.1415	20	ERR3269054
ERR3269056_CD_2014	2014	DRC	Uvira	ST515	−3.38413	29.1415	20	ERR3269056
ERR3269057_CD_2014	2014	DRC	Fizi-Baraka	ST515	−4.30058	28.94212	12	ERR3269057
ERR3269058_CD_2014	2014	DRC	Fizi-Baraka	ST69	−4.30058	28.94212	12	ERR3269058

V. cholera name	Year	Location, country or sea	Location, region/ locality	Lineage	Latitude	Longitude	Location on map†	SRA ID
ERR3269059_CD_2014	2014	DRC	Goma	ST515	−1.6835	29.2356	17	ERR3269059
ERR3269060_CD_2014	2014	DRC	Fizi-Baraka	ST515	−4.30058	28.94212	12	ERR3269060
ERR3269061_CD_2014	2014	DRC	Fizi-Baraka	ST69	−4.30058	28.94212	12	ERR3269061
ERR3269062_CD_2014	2014	DRC	Goma	ST69	−1.6835	29.2356	17	ERR3269062
ERR3269063_CD_2014	2014	DRC	Fizi-Baraka	ST613	−4.30058	28.94212	12	ERR3269063
ERR3269064_CD_2014	2014	DRC	Fizi-Baraka	ST612	−4.30058	28.94212	12	ERR3269064
ERR3269065_CD_2015	2015	DRC	Goma	ST515	−1.6835	29.2356	17	ERR3269065
ERR3269066_CD_2015	2015	DRC	Goma	ST515	−1.6835	29.2356	17	ERR3269066
ERR386629_CD_2009	2009	DRC			−5.91312	29.20005	4	ERR386629
ERR386661_ZM_2012	2012	DRC						ERR386661
ERR386712_CD_2012	2012	DRC	Biyela	ST515	−4.32153	15.31185	5	ERR386712
ERR572559_CD_2013	2013	DRC	Nyemba	ST69	−8.16966	25.38507	1	ERR572559
ERR572810_CD_2001	2001	DRC	Ankoro		−6.75053	26.94274	2	ERR572810
ERR976553_UG_1998	1998	Uganda						ERR976553
ERR976558_KM_1998	1998	Comoros						ERR976558
ERR976569_RW_1998	1998	Rwanda						ERR976569
ERR976575_SD_1998	1998	Sudan						ERR976575
ERR976593_MG_2000	2000	Madagascar						ERR976593

*DRC, Democratic Republic of the Congo.
†Map in Figure 1.

Appendix Table 2. Results of MLST analysis of DRC *V. cholerae* strains

Sample name	MLST profile (ST)
AGC_12_CD_2016	69
AGC_13_CD_2016	69
AGC_9_CD_2016	69
AGC_1_CD_2015	515
AGC_10_CD_2016	515
AGC_11_CD_2016	515
AGC_14_CD_2017	515
AGC_15_CD_2017	515
AGC_16_CD_2017	515
AGC_17_CD_2017	515
AGC_18_CD_2017	515
AGC_19_CD_2017	515
AGC_2_CD_2015	515
AGC_20_CD_2017	515
AGC_21_CD_2017	515
AGC_22_CD_2017	515
AGC_23_CD_2017	515
AGC_24_CD_2017	515
AGC_3_CD_2015	515
AGC_4_CD_2015	515
AGC_5_CD_2015	515
AGC_6_CD_2015	515
AGC_7_CD_2015	515
AGC_8_CD_2015	515

Appendix Table 3. ICP1 phage which were sequenced

Strain	Isolation date	Province/Location	SRA ID
DRC32	1/11/2017	Rutshuru/Hgr	SRR18305671
DRC48	3/18/2017	Goma/Hgr	SRR18305670
DRC55	3/30/2017	Rutshuru/Tongo	SRR18305669
DRC72	3/2/2017	Kirotshe/Sake	SRR18305668
DRC74	3/2/2017	Kirotshe/Sake	SRR18305667
DRC82	4/11/2017	Rutshuru/Ntamugenga	SRR18305666
DRC87	4/15/2017	Nyiragongo/Kibumba	SRR18305665
DRC106	4/22/2017	Kibumba	SRR18305664

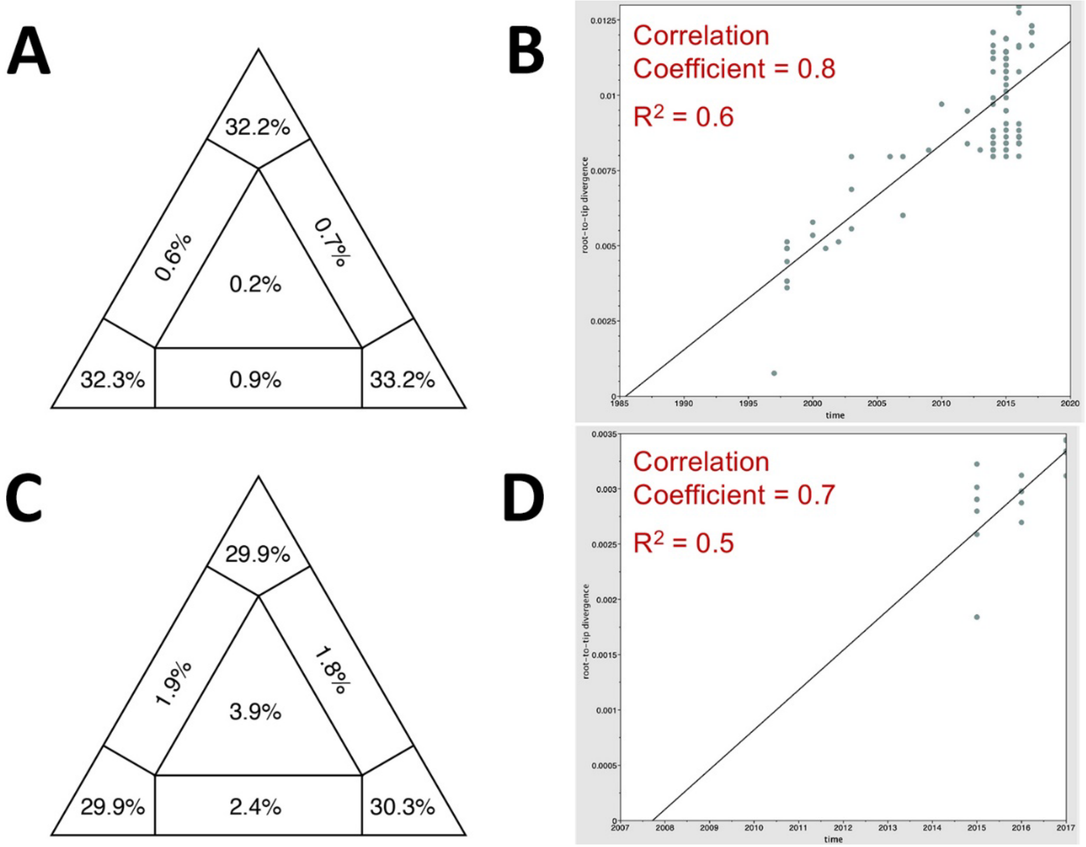

Appendix Figure 1. Estimations of phylogenetic and temporal signal from the DRC phylogenies. Presence of phylogenetic signal in the dataset of the *V. cholerae* A) dataset displayed in Figure 1 (isolates collected by our group and downloaded from NCBI), as well as C) the dataset displayed in Figure 3 (isolates collected by our group only), was evaluated by likelihood mapping check for alternative topologies (tips), unresolved quartets (center) and partly resolved quartets (edges) for each dataset. Linear regressions of root-to-tip genetic distance within the ML phylogeny (tree in Figure 1) against sampling time for each taxon. Temporal resolution for B) dataset displayed Figure 1 or D) dataset displayed in Figure 3 was assessed using the slope of the regression, with positive slope indicating sufficient temporal signal. Correlation coefficient, r, and R^2 are reported in the temporal signal plot.

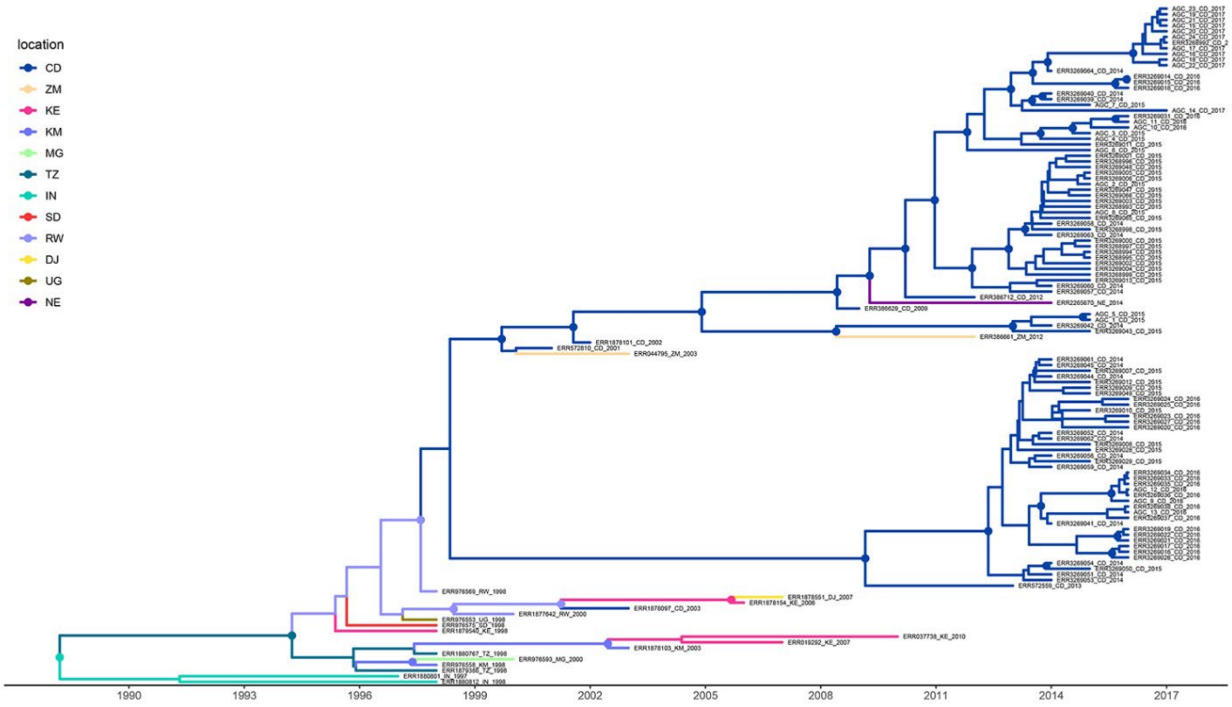

Appendix Figure 2. MCC in Figure 1 tree with tips. Phylogeny reported in Figure 1 with tips. Branches of the phylogeny are scaled in time and colored by country of origin as shown in the legend (location). Circles in internal node indicate posterior probability support >0.9 and the colors indicate the ancestral country inferred by Bayesian phylogeography reconstruction. CD, Democratic Republic of Congo; ZM, Zambia; KE, Kenya; KM, Comoros; MG, Kyrgyzstan; TZ, United Republic of Tanzania; IN, India; SD, Sudan; RW, Rwanda; DJ, Djibouti; UG, Uganda; NE, Niger

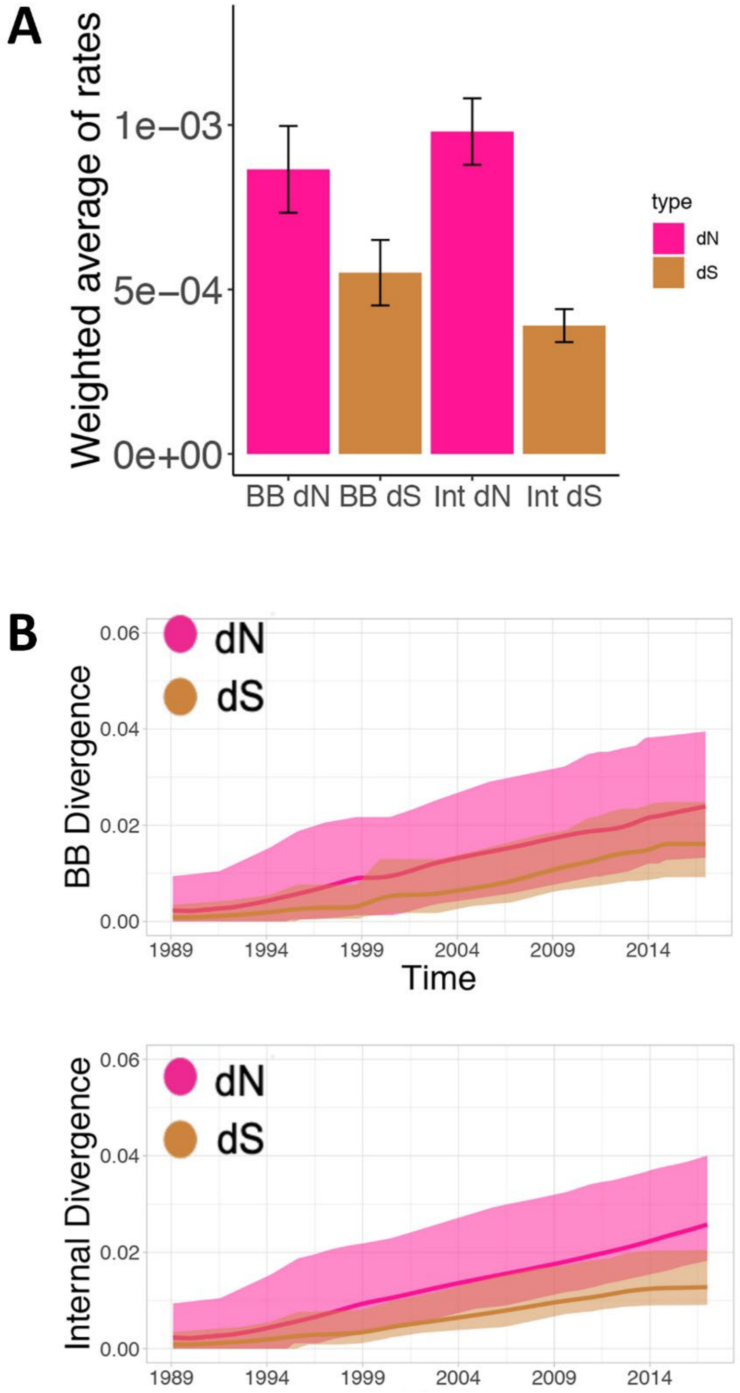

Appendix Figure 3. Tree, synonymous and nonsynonymous substitution rates. A) Weighted average of synonymous and nonsynonymous substitution rates of backbone and internal branches based on the Bayesian phylogeography tree in Figure 1. B) Absolute synonymous (tan) and nonsynonymous (pink) divergence rates (y-axis) against time in years (x-axis) of backbone (top) and internal branches (bottom) of the phylogeny. BB, backbone; dN, nonsynonymous substitution rates; dS, synonymous substitution rates
